# Hypoglycemic effect of camel milk powder in type 2 diabetic patients: A randomized, double‐blind, placebo‐controlled trial

**DOI:** 10.1002/fsn3.2420

**Published:** 2021-06-29

**Authors:** Yajie Zheng, Fang Wu, Ming Zhang, Bing Fang, Liang Zhao, Lijie Dong, Xiaojuan Zhou, Shaoyang Ge

**Affiliations:** ^1^ School of Food Food and Health Beijing Technology and Business University Beijing China; ^2^ Department of Nutrition and Health China Agricultural University Beijing China; ^3^ Beijing Chinese Medicine Hospital Pinggu Hospital Beijing China; ^4^ Key Laboratory of Functional Dairy Co‐constructed by Ministry of Education and Beijing Government, and Beijing Laboratory of Food Quality and Safety China Agricultural University Beijing China; ^5^ Hebei Engineering Research Center of Animal Product Sanhe China

**Keywords:** adipokines, appetitive hormones, gut microbiota, inflammation, lipid profile, myokines

## Abstract

Fresh camel milk was widely accepted to help to prevent and control of diabetes, especially in Africa, Middle East, and cooler dry areas of Asia. In this study, type 2 diabetic patients were enrolled to supplement with 10 g of camel milk powder twice a day for 4 weeks (*n* = 14), cow milk powder served as the placebo (*n* = 13). It was found that camel milk supplement decreased fasting blood glucose, 2‐hr postprandial blood glucose, serum content of total cholesterol, resistin, and lipocalin‐2. There was also a significant increase in serum content of osteocrin, amylin, and GLP‐1in camel milk group, indicating an improvement on adipose tissue and skeletal muscle. Camel milk powder supplement significantly enriched the relative abundance of *Clostridium_sensu_stricto_1* and *[Eubacterium]_eligens_group* compared with cow milk after the 4‐week intervention. This study suggested that camel milk powder can be used as a functional food help to treat type 2 diabetes.

## Graphical abstract

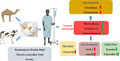

## 1 INTRODUCTION

Diabetes is a common chronic disorder and metabolic disease in which body cannot effectively control glucose homeostasis. Diabetes can lead to severe damage to the heart, eyes, kidneys, blood vessels, and nerves. According to the data of the International Diabetes Federation, it is estimated that 463 million people have diabetes in 2019 and this number is projected to reach 578 million by 2030 and 700 million by 2045 (Agrawal et al., 2011; Thomas et al., 2019). China, which is the most populous country, ranks number one with an estimate of 109.6 million adults with diabetes (Hu & Jia, 2018). Type 2 diabetes mellitus (T2DM) is a widely prevalent form, classified by metabolic disorders with hyperglycemia, and considered as a main health concerned issue comprising higher morbidity and mortality. As a result, more and more studies focused on the development of antidiabetic medications (Bailey et al., 2016; Delzenne et al., 2015) and functional foods (Beidokhti & Jager, 2017; Martel et al., 2017; Naveen & Baskaran, 2018), especially for the treatment of T2DM which accounts for about 90% of the diabetes and is preventable and can be cured by medication and health lifestyle (Thomas et al., 2019).

Over the years, many types of traditional food treatments and natural remedies have been used to treat diabetes (Leiherer et al., 2013; Rudkowska, 2009); however, the validity and effectiveness of just a few of them have been evaluated. There is a traditional belief in Africa, Asia, and Middle East that regular consumption of camel milk may aid in prevention and control of diabetes, and epidemiologic study also reported a significant lower incidence of diabetes in people consuming camel milk than those did not have the habit in the same community (0.4% vs. 5.5%) (Agrawal, Budania, et al., 2007; Agrawal, Saran, et al., 2007). The hypoglycemic function of camel milk has been confirmed in streptozotocin‐diabetic rats (Abdel‐Salam & Al‐Damegh, 2018; Al‐Numair, Chandramohan, & Alsaif, 2011a, 2011b; Korish, 2014; Wang et al., 2009), type 1 diabetes (Agrawal et al., 2005, 2011; Agrawal et al., 2003a, 2003b; Agrawal, Budania, et al., 2007; Agrawal, Saran, et al., 2007; Mohamad et al., 2009), and T2DM (Agrawal et al., 2011; Ejtahed et al., 2015; Korish, 2014; Wang et al., 2009) characterized by reduced fasting glucose and glycosylated hemoglobin. Insulin‐like and whey protein hydrolysates may be the main ingredients in hypoglycemic effect of camel milk. Insulin‐like was proved by the decreased insulin requirement in type 1 diabetic patients (Agrawal et al., 2011; Agrawal et al., 2003a, 2003b; Mohamad et al., 2009). However, whether these proteins can resist the digestion in the gastrointestinal protease was still a matter of debate. Another probable active component for hypoglycemic effects may be whey protein or its hydrolysate. Camel whey protein was also found to have an inhibitory effect on dipeptidyl peptidase IV (Ayoub et al., 2018; Mudgil et al., 2018), which control the secretion of insulin. It also reported that the hydrolysate of camel whey protein harbored greater antioxidant and immunomodulatory activities than bovine and other whey proteins (Ayoub et al., 2018; Badr, Ramadan, et al., 2017).

Camel mainly lives in the desert areas of Africa/Middle East or the cooler dry areas of Asia (Brezovecki et al., 2015), leading to the unavailability of fresh camel milk for people lived in other areas. However, all the clinical trials were based on the uptake of fresh camel milk, whereas the antidiabetic effect of processed camel milk products such as camel milk powder was lack of study. Therefore, the aim of this study was to evaluate the antidiabetic effect of camel milk powder in T2DM patients. Given that T2DM patients have an increased cardiovascular risk, and may develop complications such as diabetic foot, the protective effects of probiotic camel milk on lipid profile, inflammatory cytokines, myokines, and adipokines in T2DM patients were also evaluated.

## 2 MATERIALS AND METHODS

### 2.1 Participants and study design

We performed a double‐blind, randomized, placebo‐controlled pilot study over 4 weeks to investigate the effects of camel milk in type 2 diabetic patients. Type 2 diabetic patients were recruited from subjects attending to the clinic of Beijing Chinese Medicine Hospital Pinggu Hospital. Inclusion criteria were age 35–68 years, absence of gastrointestinal disease, and willingness to abstain from intake of other milk, probiotic food, and fermented milk products during the study but otherwise stick to previous eating habits. Exclusion criteria were pregnancy or lactating in women, cancer, allergy, or intolerance to camel milk or cow milk.

After run‐in phase, participants were categorized into two groups and received camel milk powder and cow milk powder (placebo control), respectively, for 4 consecutive weeks (two times per day, 10 g each time). All the samples were provided by Sanhe Fucheng Biological Technology Co. Ltd (Langfang, China). Nutrient content of the camel milk and cow milk powder was shown in Table 1. Blood glucose, lipid profile, serum cytokines content, and collection of fecal samples were performed at the end of the run‐in phase and at end of the study. During the study, participants underwent interviews regarding adverse effects, symptoms, or changes in quality of life every week. The study was approved by the local ethics committee of China Agricultural University (CAUHR‐2018026) and registered at ClinicalTrials.gov (NCT04296825). All participants provided written informed consent.

**TABLE 1:** Nutrient content of the camel milk and cow milk powder

Variables	Camel milk powder	Cow milk powder
Fat (%)	32.6	28.2
Total protein (%)	30.3	25.1
‐Whey protein (%)	8.0	4.5
‐Casein (%)	20.8	20.2
Carbohydrate (%)	37.1	42.0
H_2_O (%)	4.3	2.1

### 2.2 Blood sample collection and plasma glucose measurements

Human peripheral blood was collected in Vacutainer tubes and (Cat #368921, BD Biosciences) and Vacutainer heparin tubes (Cat #367886, BD Biosciences), respectively. Blood samples were centrifuged at 1,500 × g for 30 min at room temperature and samples in for fasting glycemia, 2‐hr postprandial glycemia, insulin, and lipid measurements within 1 hr after blood collection, and serum samples in Vacutainer heparin tubes were carefully removed, aliquoted, snap‐frozen in liquid nitrogen, and stored in aliquots at −80°C until further analysis.

### 2.3 Biochemical indexes measurements

Serum insulin were measured using the Architect i2000SRanalyzer (Abbott Diagnostics), and serum content of glucose, uric acid, total cholesterol (TC), total triglyceride (TG), high‐density lipoprotein cholesterol (HDL‐C), and low‐density lipoprotein cholesterol (LDL‐C) were measured using a Roche cobas^®^ e 411 analyzer (Roche) according to the manufactures’ protocol by the certified core clinical laboratory at the Beijing Chinese Medicine Hospital Pinggu Hospital. To evaluate insulin resistance, the HOMA‐IR formulation was applied (Wallace et al., 2004) according to the following equation: HOMA‐IR = Fasting blood glucose (mmol/L) × fasting inulin concentration (μU/ml) × 18/405.

### 2.4 Gut hormones and cytokines assays

For determination of appetitive hormones (amylin [active], ghrelin [active], glucagon‐likepeptide 1 [GLP‐1, active], and pancreatic polypeptide [PP]), inflammation cytokines (TNF‐α, IL‐6, MCP‐1), myokines (FGF‐21, irisin, osteocrin, osteonectin), and adipokines (adiponectin, resistin, lipocalin‐2,adipsin), human cytokine immunobead panels (Milliplex, Millipore, Cat # MHEMAG‐34K, HMYOMAG‐56K, and HADK1MAG‐61K‐04) coupled with a multiplex assay (involving xMAP technology, Luminex) were used according to the manufactures’ protocol.

### 2.5 Gut microbiota analysis

A total of 54 cecal samples were collected at the end of the run‐in phase and at end of the study. All samples were collected into sterile tubes containing RNA later and stored at −20°C. DNA was extracted from fecal samples using the phenol–chloroform extraction method (Köchl, 2005) and quantified using a Nano Drop spectrophotometer (OneC, Thermo Fisher Scientific) and stored at −80°C until further analysis.

DNA was amplified using the universal primers 338F (5′‐ ACTCCTACGGGAGGCAGCAG‐3′) and 806R (5′‐ GGACTACHVGGGTWTCTAAT‐3′) to target the V3‐V4 region of bacterial 16S rRNA. The resulting 468‐bp‐sized products were assessed, quantified, pooled, and sequenced on an Illumina Miseq PE300 platform (Illumina) at Shanghai Majorbio Bio‐pharm Technology Co. Ltd. (Shanghai, China) using a paired‐end sequencing strategy. Raw data were spiced, filtered, and then used to select the operational taxonomic units (OTUs) with USEARCH software (version 7.0) and a default cutoff of 97% sequence similarity.

Operational taxonomic units were further subjected to the Ribosomal Database Project classifier software for taxonomic identification with an 80% confidence threshold at the phylum, class, order, family, genus, and species levels. Further analysis such as abundance and pairwise comparison of gut microbiome was analyzed on the platform of Majorbio I‐Sanger Cloud Platform (www.i‐sanger.com). Pairwise comparison of the microbiome communities of the different groups was performed at genus levels.

### 2.6 Statistical analysis

Statistical significance was determined by paired two‐tailed Student's test analysis for end and baseline comparison and one‐way ANOVA tests for differences between treatments using GraphPad Prism version 7.0 software (San Diego, CA, USA). *p* < .05 was considered statistically significant.

## 3 RESULTS AND DISCUSSION

### 3.1 Study population

**FIGURE 1:**
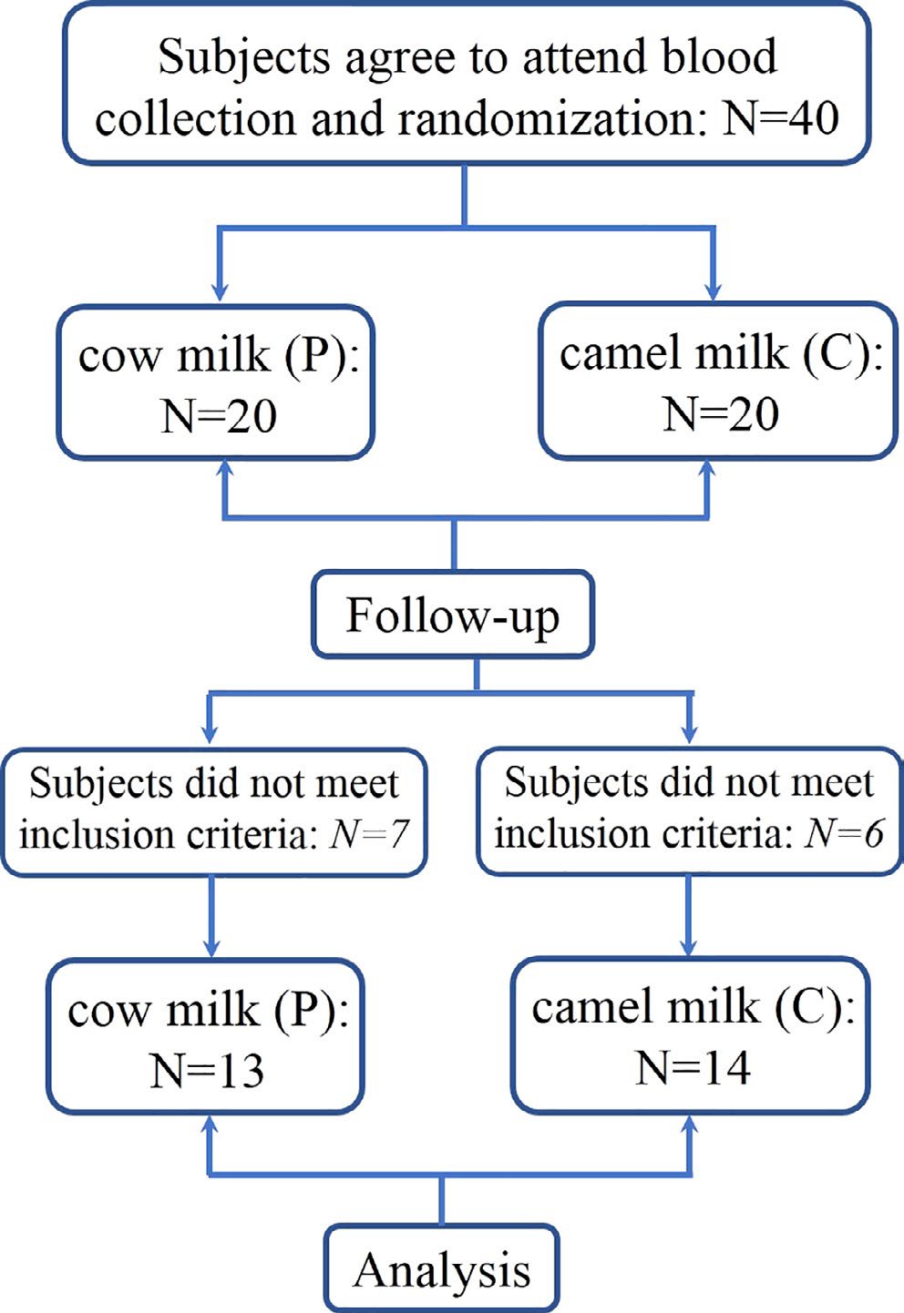
Overview of study design

Of the 40 participants that were screened and randomized, 13 persons did not pass the inclusion criteria or did not finish the experiment. The most common reasons for drop out included loss to follow‐up and poor compliance (Figure 1). At the end of the intervention, 27 participants completed the experiment and subjected to the analysis, 14 received camel milk powder (C) and 13 received cow milk powder (P) after breakfast and dinner for 4 weeks. Baseline comparison showed no significant differences in both groups (*p* > .05, Table 2). None of the participants reported any adverse effects including gastrointestinal disorders.

**TABLE 2:** Baseline characteristics of study participants

	Cow milk (P)	Camel milk (C)
*N* (female/male)	13 (9/4)	14 (8/6)
Age (years)	56.31 ± 8.15	57.29 ± 7.57
Body Mass index (BMI, kg/m^2^)	24.59 ± 2.89	26.56 ± 4.40
Waist Hip Rate (WHR)	0.94 ± 0.05	0.93 ± 0.06
Body fat (%)	32.28 ± 5.21	33.57 ± 7.70
Visceral fat (cm^2^)	111.23 ± 28.84	111.23 ± 28.84
Fasting glycemia (mmol/L)	9.33 ± 1.41	10.48 ± 3.78
2‐hr Postprandial glycemia (mmol/L)	13.17 ± 2.11	16.19 ± 5.28
Insulin (μU/ml)	8.43 ± 2.87	11.01 ± 7.11
Uric acid (μmol/L)	353.67 ± 81.15	318.66 ± 78.64
TG (mmol/L)	4.79 ± 0.59	5.10 ± 1.00
TC (mmol/L)	1.53 ± 0.67	1.14 ± 0.14
HDL‐C (mmol/L)	1.27 ± 0.17	1.22 ± 0.26
LDL‐C (mmol/L)	2.95 ± 0.59	3.33 ± 0.86
TC/HDL‐C	3.83 ± 0.76	4.47 ± 0.77

Note

Data were analyzed for normal distribution by SPSS and expressed as mean ± S.D.

### 3.2 Changes in glycemic indices and serum insulin

**FIGURE 2:**
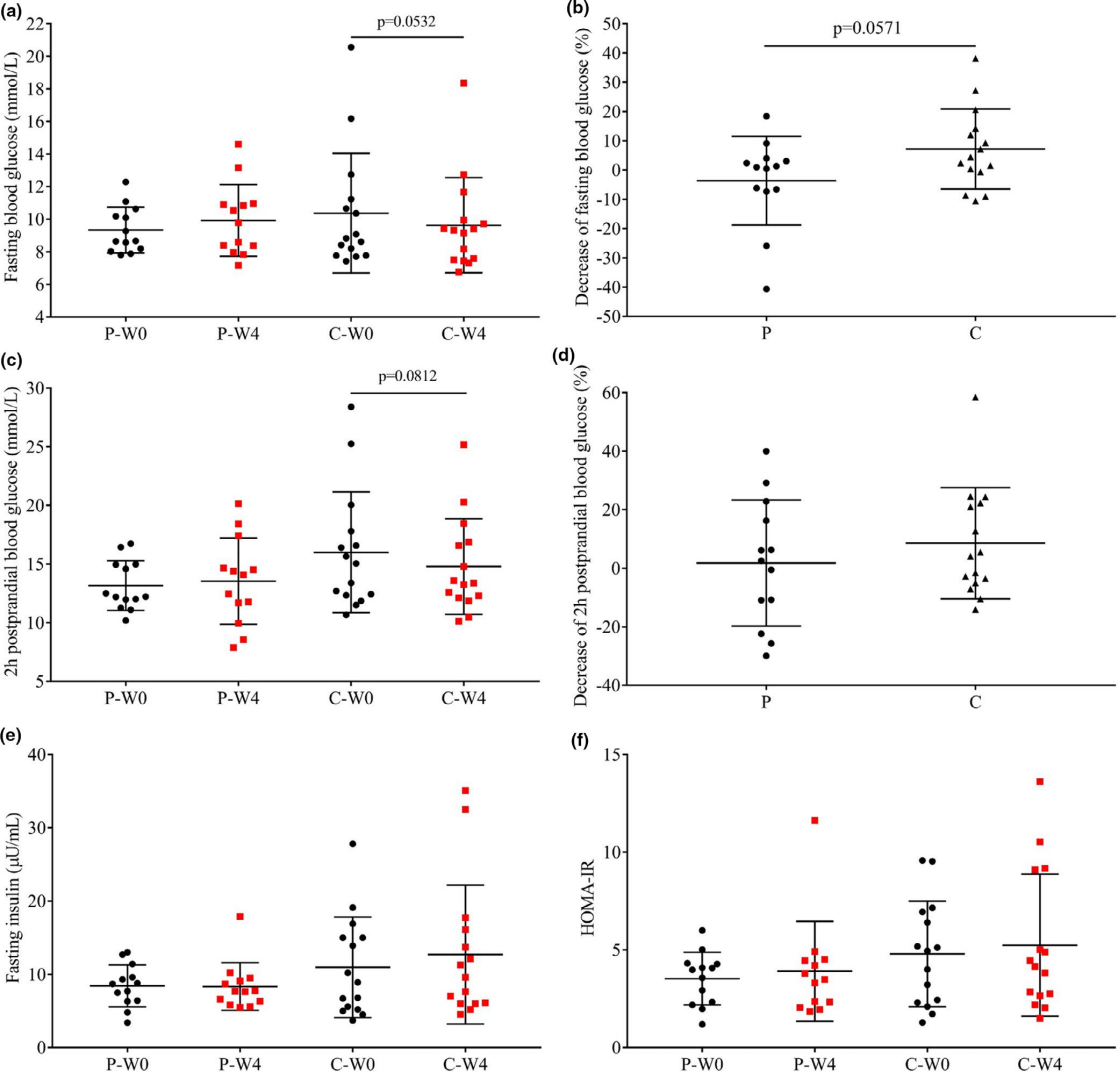
Fasting blood glucose, 2‐hr postprandial blood glucose, and fasting serum insulin and HOMA‐IR in each group before (W0) and after (W4) the intervention. (a) fasting blood glucose; (b) decrease of fasting blood glucose; (c) 2‐hr postprandial blood glucose; (d) decrease of 2‐hr postprandial blood glucose; (e) fasting serum insulin; and (f) HOMA‐IR in patients intervened with cow milk (Group P, placebo) and camel milk (Group C)

Fasting blood glucose, 2‐hr postprandial blood glucose, and fasting serum insulin of patients before and after 4‐week intervention were shown in Figure 2. At baseline, there were both no significant differences between groups (*p* > .05). After the intervention, patients in the group C exhibited an obvious decrease in fasting blood glucose (*p* = .0532, Figure 2a). There was also an nonsignificant decrease in 2‐hr postprandial blood glucose after the intervention (Figure 2c).

Previous studies about the hypoglycemic effect of camel milk were mainly based on patients with type I diabetes mellitus (Agrawal et al., 2005, 2011; Agrawal, Budania, et al., 2007; Agrawal, Saran, et al., 2007; Agrawal et al., 2003a, 2003b; Mohamad et al., 2009) or streptozotocin‐induced diabetic rats (Al‐Numair et al., 2011a; Khan et al., 2013; Wang et al., 2009). There was a consistent significant decrease in fasting blood glucose when treating type I diabetes mellitus; however, the result in type II diabetes mellitus patients was inconsistent (Agrawal et al., 2003a, 2003b; Brezovecki et al., 2015). In this study, there was a decrease in fasting blood glucose (*p* = .0532, Figure 2a) in patients intervened with camel milk (group C).

It was reported that one of the potential molecular basis of the antidiabetic properties of camel milk was related to the higher insulin content and insulin‐like amino acid composition of whey proteins (Beg et al., 1986). Although only few quantitative studies about the insulin content of camel milk were reported (Wernery et al., 2006), but evidence for the significant reduction in insulin dose required in type I diabetic patients still to be brought (Agrawal et al., 2005, 2011; Agrawal, Budania, et al., 2007; Agrawal, Saran, et al., 2007; Agrawal et al., 2003a, 2003b; Mohamad et al., 2009; Wang et al., 2009). In this study, serum content of insulin was not affected (Figure 2e *p* > .05) Probiotics intervention did not improve the insulin resistance (HOMA‐IR) of the patients (Figure 2f, *p* > .05).

Our result is in accordance with previous studies in type I diabetic patients (Agrawal et al., 2005, 2011; Agrawal, Budania, et al., 2007; Agrawal, Saran, et al., 2007; Agrawal et al., 2003a, 2003b; Mohamad et al., 2009; Wang et al., 2009). It was reported that the antidiabetic activity of camel milk is mediated by an insulin‐like effects on beta‐cells of the pancreas (Abdulrahman et al., 2016), which may lead to the different results between type I and type II diabetes mellitus. As expected, the changes in plasma insulin were inconsistency in previous type II diabetes mellitus (Agrawal et al., 2003a, 2003b; Brezovecki et al., 2015).

### 3.3 Changes in lipid profile and cardiovascular risk

**FIGURE 3:**
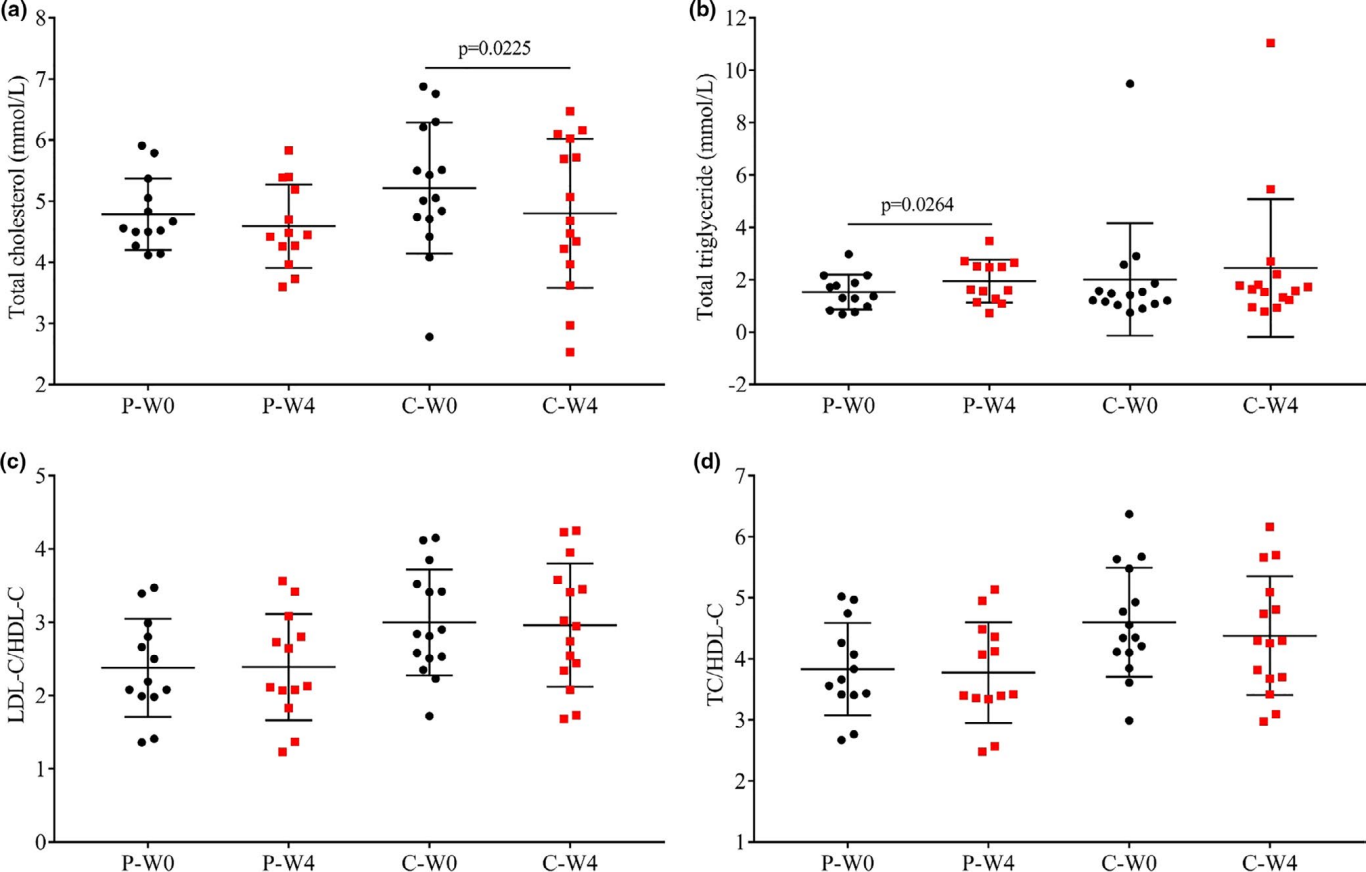
Lipid profile in each group before (W0) and after (W4) the intervention. (a) total cholesterol; (b) total triglyceride; (c) the LDL cholesterol/HDL cholesterol ratio; (d) total cholesterol/HDL cholesterol ratio in patients intervened with cow milk (Group P, placebo) and camel milk (Group C)

Total cholesterol, total triglyceride, and the indicators of vascular risk (LDL/HDL cholesterol ratio and TC/HDL‐C) were shown in Figure 3, and at baseline and postintervention, there were both no significant differences between groups (*p* > .05). After a 4‐week intervention, the total cholesterol of patients in group C decreased (*p* = .0225, Figure 3a), although there were no significant effects in the total triglyceride, patients in the placebo group (Group P) had a significantly higher total triglyceride (*p* = .0264, Figure 3b). There were no significant changes in LDL‐C/HDL‐C before and after the interventions (*p* > .05, Figure 3c), but an nonsignificant decrease in the atherogenic index in group C (TC/HDL‐C, Figure 3d).

The effect of camel milk on lipid profile was mainly studied in diabetic rats (Khan et al., 2013; Korish, 2014; Wang et al., 2009), which was consistent with our findings that there was a decrease in total cholesterol after the intervention in group C (*p* = .0225, Figure 3a). However, in the limited studies in type II diabetic patients, one study (Wang et al., 2009) was in accordance with ours while another one reported that there were no changes in lipid profile (Ejtahed et al., 2015). Furthermore, there was also a decrease in the ratio of total cholesterol and HDL‐C (Figure 3d), indicating a decreased vascular risk by camel milk. It was interested to see the significant increase in total triglyceride in group P, the similar effect was found in alloxan‐induced diabetic dogs with a significant increase total cholesterol when treated with cow milk for 5 weeks (Sboui, Djegham, et al., 2010; Sboui, Khorchani, et al., 2010).

### 3.4 Changes in inflammatory cytokines

**FIGURE 4:**
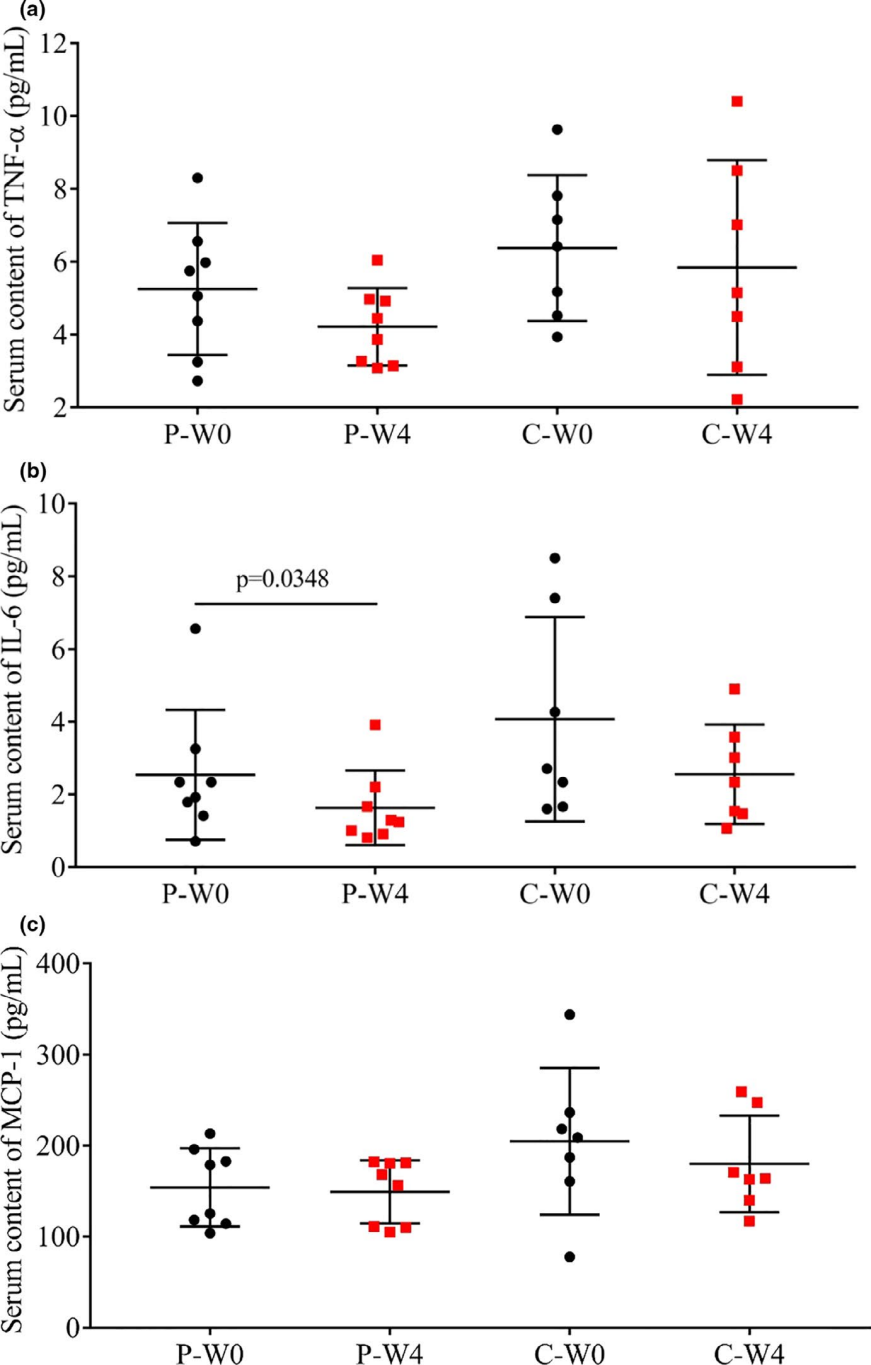
Serum contents of inflammatory cytokines in each group before (W0) and after (W4) the intervention. (a) TNF‐α; (b) IL‐6; (c) MCP‐1 in patients intervened with cow milk (Group P, placebo) and camel milk (Group C)

Serum contents of inflammatory markers (TNF‐α, IL‐6, MCP‐1) in patients before and after 4‐week intervention were shown in Figure 4. After the 4‐week intervention, there were an nonsignificant decrease in the content of IL‐6 in group C, which was significant in patients in group P (*p* = .0348, Figure 4b). Furthermore, the decrease in TNF‐α (Figure 4a) and MCP‐1 (Figure 4c) was also more obvious in group P than in group C.

It was reported that the antidiabetic mechanism of camel milk may be also related to its greater antioxidant and immunomodulatory activities than bovine and other whey proteins (Ayoub et al., 2018; Badr, Ramadan, et al., 2017). Meanwhile, it is widely accepted that defects in both redox and the immune systems resulted in the damage and the destruction of the pancreatic beta‐cells and linked to diabetes mellitus (Newsholme et al., 2019). And the treatment of diabetic rats with camel milk or camel milk whey proteins was shown to reduce the proinflammatory IL‐1β, IL‐6, and TNF‐α (Badr, Sayed, et al., 2017; Korish, 2014; Mahmoud et al., 2016). Here in our study, we only found a significant decrease in IL‐6 (*p* = .0103, Figure 4b).

### 3.5 Changes in adipokines and myokines profile

**FIGURE 5:**
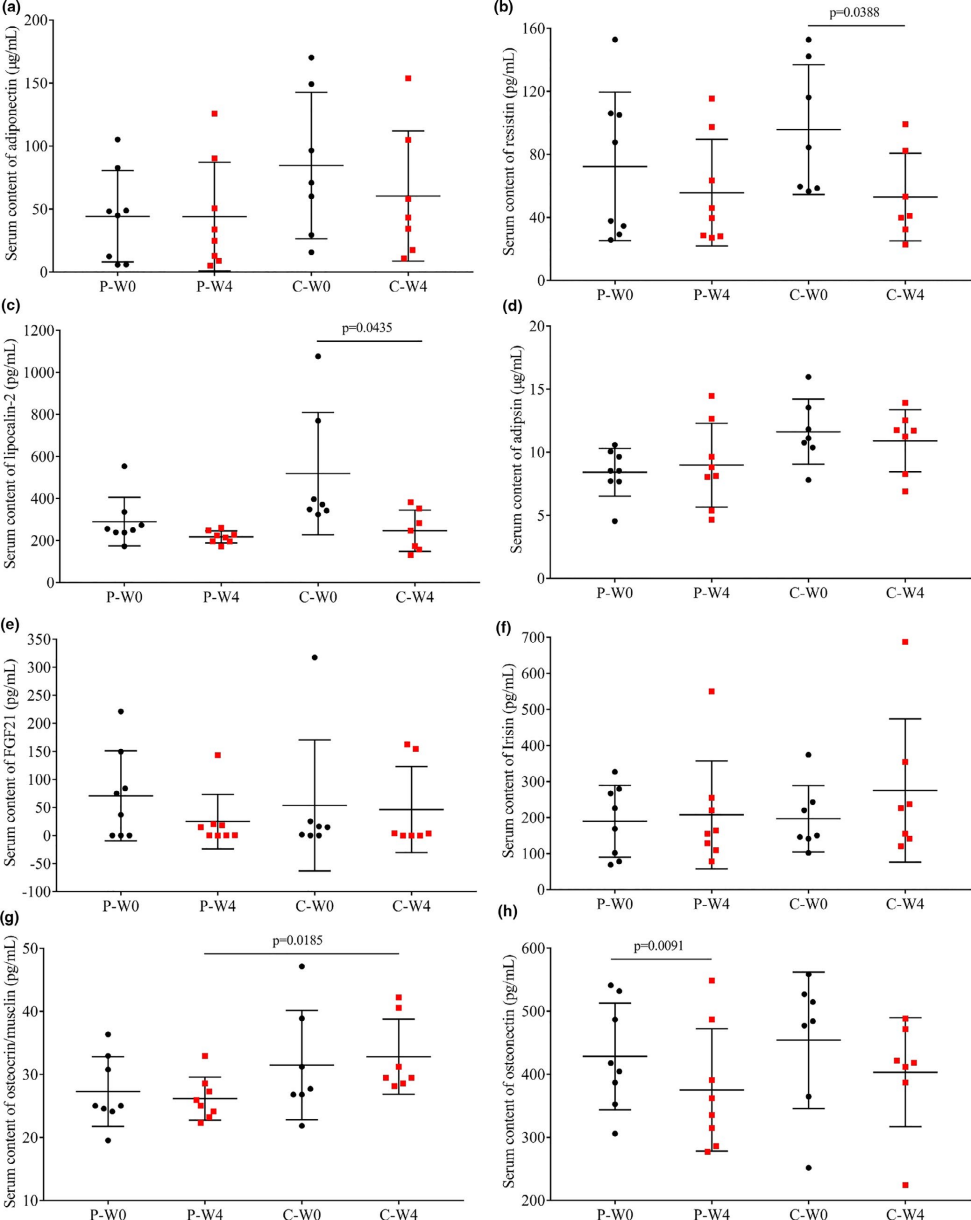
Serum contents of adipokines and myokinesin each group before (W0) and after (W4) the intervention. (a) adiponectin; (b) resistin; (c) lipocalin‐2; (d) adipsin; (e) FGF‐21; (f) irisin; (g) osteocrin; (h) osteonectin in patients intervened with cow milk (Group P, placebo) and camel milk (Group C)

Serum contents of adipokines (adiponectin, resistin, lipocalin‐2, adipsin) and myokines (FGF‐21, irisin, osteocrin, osteonectin) in patients before and after 4‐week intervention were shown in Figure 5. There was only a significant decrease in serum content of resistin (*p* = .0388, Figure 5b) and lipocalin‐2 (*p* = .0435, Figure 5c) in patients of group C. Interestingly, a significant decrease in serum content of osteonectin was found in group P (*p* = .0091, Figure 5h).

Recent evidence has identified skeletal muscle and adipocytes as secretory organs, which communicate with each other to regulate energy homeostasis and insulin sensitivity though the cytokines called myokines and adipokines (Galic et al., 2010; Pedersen & Febbraio, 2012), respectively. As we can see from Figure 5, intervention with camel milk significantly decreased the content of resistin and lipocalin‐2 levels, which was reported to be good for the improving of diabetes. Elevated serum lipocalin‐2 is closely and independently associated with impaired glucose regulation and type 2 diabetes in Chinese people (Huang et al., 2012), and the lipocalin‐2 deficiency attenuates insulin resistance associated with obesity in mice (Law et al., 2010). Resistin promotes insulin resistance in mice, whereas whether it does so in humans is unclear (Heilbronn et al., 2004; Steppan et al., 2001) because it was synthesized in adipocytesin mice whereas in humans it is generated by macrophages and monocytes, but not adipocytes (Oh et al., 2017). According to our results, there was a significant decrease in resistin after the intervention accompanied by the decreased fasting blood glucose, and it maybe also positively correlated with the hyperglycemia. There were no significant changes in myokines when compared serum contents before and after the intervention (*p* > .05); however, there was a significant elevation in osteocrin in patients intervened with camel milk than those took cow milk (Figure 5g, C‐W4 vs. P‐W4, *p* = .0185), which is an indicator for the improvement in skeletal muscle (Subbotina et al., 2015). Furthermore, intervention with cow milk significantly decreased serum content of osteonectin (*p* = .0091, Figure 5h), which is concerned with normal remodeling and maintenance of bone mass (Zhu et al., 2019). The decrease may be related to the unimproved hyperglycemia.

### 3.6 Changes in appetitive hormones

**FIGURE 6:**
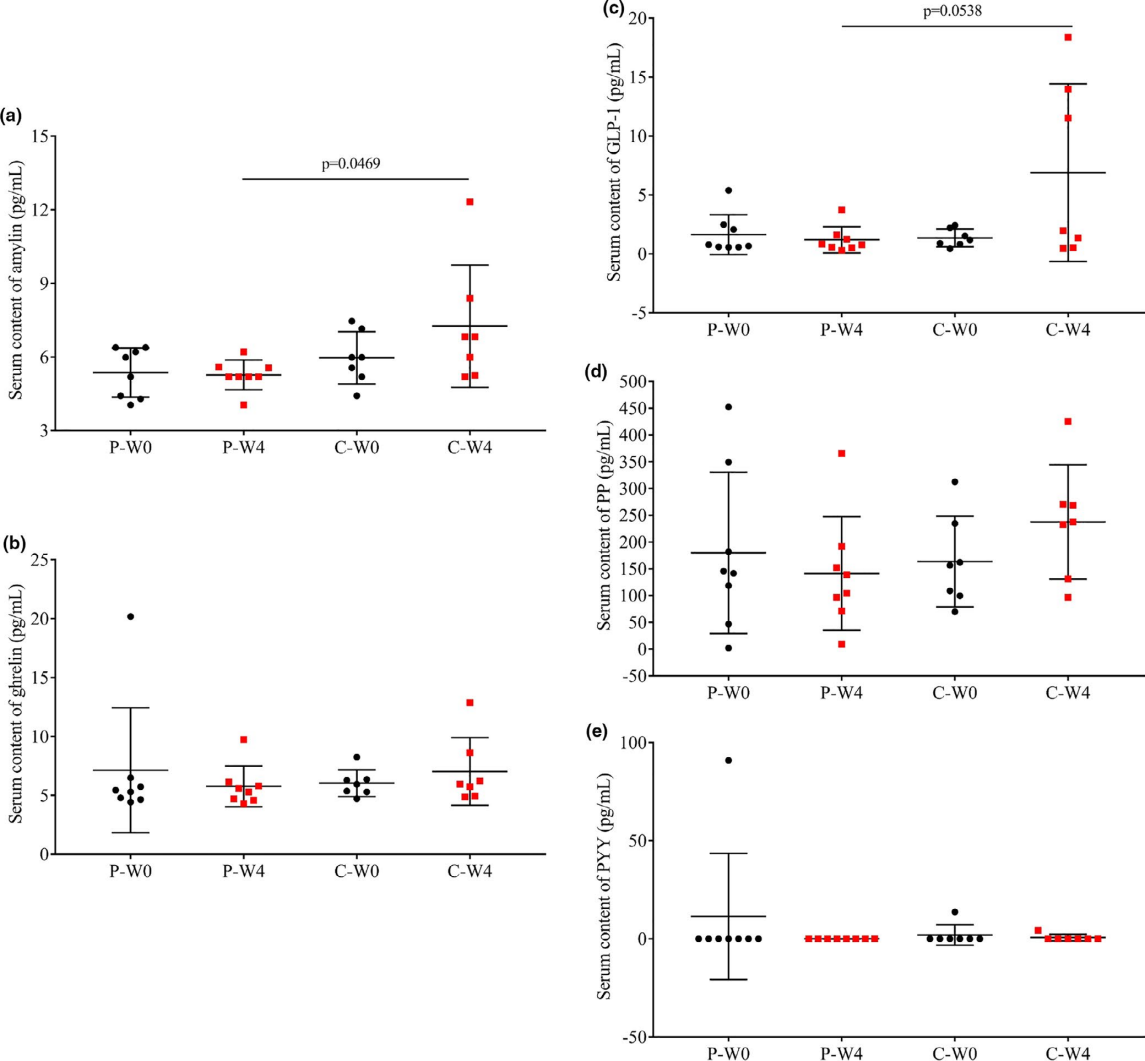
Serum contents of appetitive hormonesin each group before (W0) and after (W4) the intervention. (a) amylin; (b) ghrelin; (c) GLP‐1; (d) PP; (e) PYY in patients intervened with cow milk (Group P, placebo) and camel milk (Group C)

Serum contents of appetitive hormones (amylin, ghrelin, GLP‐1, PP, PYY) in patients before and after 4‐week intervention were shown in Figure 6. There were no significant changes before and after the intervention with either camel milk or cow milk. However, it was note‐worthy to see that there was a significant higher concentration of amylin (Figure 6a, *p* = .0469) and GLP‐1 (Figure 6c, *p* = .0538) in the serum of patients intervened with camel milk than those intervened with cow milk.

Camel whey protein was also found to have an inhibitory effect on dipeptidyl peptidase IV (Ayoub et al., 2018; Mudgil et al., 2018), the key enzyme that cleaves and inactivates incretins such as GLP‐1 and then indirectly control the secretion of insulin (Andersen et al., 2018). As we can see the changes in appetite hormones from Figure 6, there was only a nonsignificant increase of GLP‐1 and PP after the intervention of camel milk (*p* > .05, Figure 6c and d). Although the elevation of GLP‐1 was nonsignificant before and after the intervention, the increase was obvious when compared patients in different group after the intervention (C‐W4 vs. P‐W4, *p* = .0538). Amylin, a hormone colocalized, copackaged, and cosecreted with insulin from adult pancreatic islet β cells, was reported to be deficient in diabetic patients (Schmitz et al., 2004). Although the increase in amylin was nonsignificant before and after the intervention with camel milk (C‐W4 vs. C‐W0, *p* > .05), its content in patients supplemented with camel milk for 4 weeks was significantly higher than patients supplemented with cow milk (Figure 6a, C‐W4 vs. P‐W4, *p* = .0469).

### 3.7 Changes in gut microbiota

**FIGURE 7:**
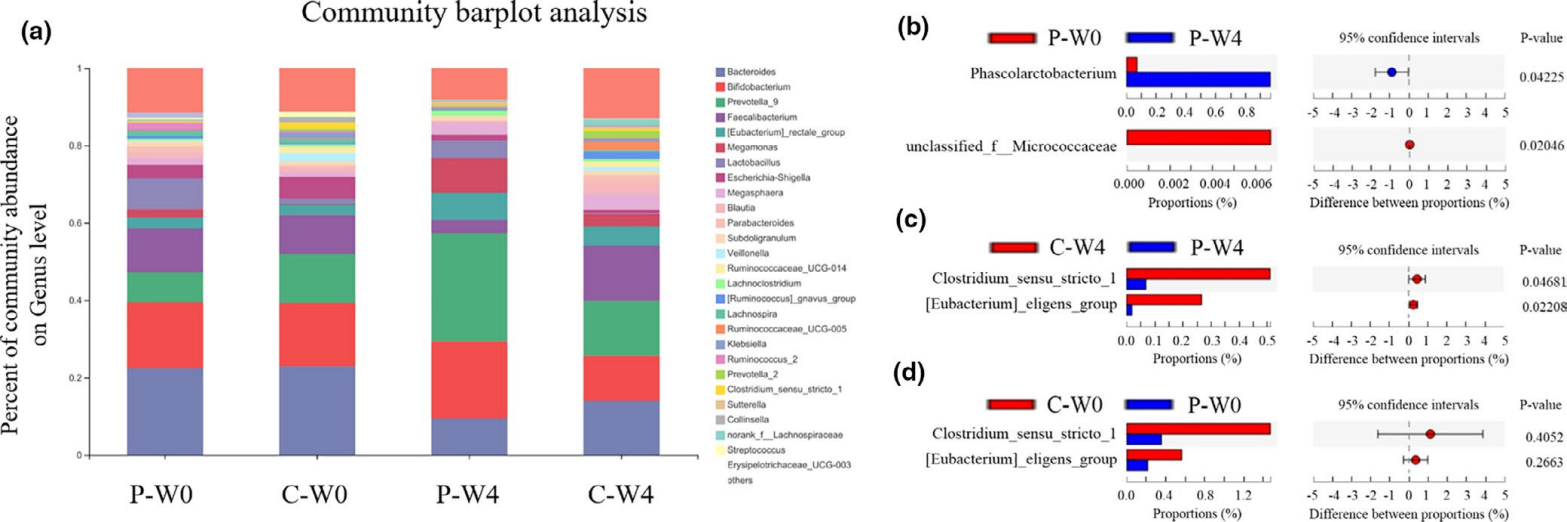
Changes o gut microbiota at the genus level in patitents intervened with cow milk (Group P, placebo) and camel milk (Group C) before (W0) and after (W4) the intervention. (a) Community abundances of gut microbiota composition. (b, c, d) Pairwise comparison of the microbiota communities of the four groups by two‐tailed Student’s t‐test

The different gut bacteria at the genus level before and after the intervention or between different groups analyzed by two‐tailed Student's *t* test were shown in Figure 7. The community abundances on genus levels of gut microbiome composition were shown in Figure 7a. The microflora composition of the group C and P was very similar before the intervention began. However, after probiotics intervention, there were some differences in flora composition. In order to further clarify the specific differences, we performed pairwise comparison of the microbiome communities of the four groups at genus levels. There were no different genera before and after the intervention in group C. But for group P, there was a significant increase in *Phascolarctobacterium* (*p* = .04225) and a decrease in unclassified_f__*Micrococcaceae* (*p* = .02046, Figure 7a). When compared gut microbiota between patients in group C and P after the 4‐week intervention, there was a significant increase in the relative abundance of *Clostridium_sensu_stricto_1* and *[Eubacterium]_eligens_group* in group C (Figure 7b, *p* < .05, C‐W0 vs. P‐W4), which was not due to the difference between individuals before the intervention (Figure 7c *p* > .05, C‐W0 vs. P‐W0).

More and more evidence suggests a close relationship between gut microbiota and diabetes (Tilg & Moschen, 2014). Phascolarctobacterium can produce short‐chain fatty acids, including acetate, propionate, and butyrate, and can be dramatically increased by berberine and metformin (Zhang et al., 2015). Fermented cow milk was reported to significantly enrich the relative abundance of Phascolarctobacterium (Rettedal et al., 2019), and there was no significant difference between the two groups both before and after the intervention (Figure S1, *p* > .05), so this was not the reason for the different effect in glycemic index between camel milk and cow milk. Clostridium_sensu_stricto_1 is one of the most important anaerobic, fermenting bacteria in human gut which may metabolize various compounds such as carbohydrates and amino acids (Wiegel et al., 2006). There was a significant enrichment in this genus in patients of group C (Figure 7b); however, its proportion decreased significantly when compared gut microbiota before and after the intervention. In addition, the other genus changed significantly between the different intervention, and [Eubacterium]_eligens_group was found at low levels in individuals with type 2 diabetes mellitus (Karlsson et al., 2013; Viciani, 2017). We can also see from Figure 7c and b that its relative abundance decreased after the intervention with both milks. Interestingly, our study also found that there was a significant lower abundance of [Eubacterium]_eligens_group in patients intervened with camel milk which exhibited an improvement in the symptom of diabetes.

## 4 CONCLUSION

In conclusion, the present work demonstrates that camel milk powder can also exhibited an antidiabetic activity in type 2 diabetic patients. After a 4‐week intervention with 10 g of camel milk powder twice a day, there was a decrease in fasting blood glucose and 2‐hr postprandial blood glucose, as well as serum content of total cholesterol. Meanwhile, supplement with camel milk powder also significantly decreased serum content of resistin and lipocalin‐2, adipokines which was reported to be positively associated with diabetes. Patients intervened with camel milk also exhibited a significant higher content of amylin and GLP‐1 than patients intervened with cow milk. These results in combination suggested that camel milk powder can be used as part of the treatment of type 2 diabetes.

## INFORMED CONSENT

Study procedure was explained for participants, and all participants provided written informed consent.

## CONFLICT OF INTEREST

The authors declare no conflict of interest.

## AUTHOR CONTRIBUTIONS

**Yajie Zheng:** Conceptualization (lead); Data curation (lead); Formal analysis (lead); Investigation (lead); Writing‐original draft (lead). **Fang Wu:** Conceptualization (equal); Data curation (equal); Investigation (equal); Writing‐original draft (equal); Writing‐review & editing (equal). **Ming Zhang:** Data curation (equal); Investigation (equal); Writing‐review & editing (equal). **Bing Fang:** Data curation (equal); Methodology (equal). **Liang Zhao:** Data curation (equal); Investigation (equal). **Lijie Dong:** Investigation (equal); Methodology (equal). **Xiaojuan Zhou:** Investigation (equal). **Shaoyang Ge:** Methodology (equal).

## ETHICAL APPROVAL

The clinical experiment was registered in Clinical Trials Protocol Registration System (ClinicalTrials.gov Identifier: NCT04296825).

## Supporting information

Figure S1

## Data Availability

All authors confirm that the data supporting the findings of this study are available within the article.

## References

[fsn32420-bib-0001] Abdel‐Salam, A. M., & Al‐Damegh, M. A. (2018). Antidiabetic and immunoprophylactic effects of camel milk filtrate and bitter gourd (*Momordica charantia*) juice against alloxan‐induced oxidative stress and diabetes in rats. International Journal of Pharmacology, 14(3), 397–406. 10.3923/ijp.2018.397.406

[fsn32420-bib-0002] Abdulrahman, A. O., Ismael, M. A., Al‐Hosaini, K., Rame, C., Al‐Senaidy, A. M., Dupont, J., & Ayoub, M. A. (2016). Differential effects of camel milk on insulin receptor signaling ‐ toward understanding the insulin‐like properties of camel milk. Frontiers in Endocrinology, 7,4. 10.3389/fendo.2016.00004 26858689 PMC4728290

[fsn32420-bib-0003] Agrawal, R. P., Beniwal, R., Kochar, D. K., Tuteja, F. C., Ghorui, S. K., Sahani, M. S., & Sharma, S. (2005). Camel milk as an adjunct to insulin therapy improves long‐term glycemic control and reduction in doses of insulin in patients with type‐1 diabetes. A 1 year randomized controlled trial. Diabetes Research and Clinical Practice, 68(2), 176–177. 10.1016/j.diabres.2004.12.007 15860247

[fsn32420-bib-0004] Agrawal, R. P., Budania, S., Sharma, P., Gupta, R., Kochar, D. K., Panwar, R. B., & Sahani, M. S. (2007). Zero prevalence of diabetes in camel milk consuming Raica community of North‐west Rajasthan, India. Diabetes Research and Clinical Practice, 76(2), 290–296. 10.1016/j.diabres.2006.09.036 17098321

[fsn32420-bib-0005] Agrawal, R. P., Jain, S., Shah, S., Chopra, A., & Agarwal, V. (2011). Effect of camel milk on glycemic control and insulin requirement in patients with type 1 diabetes: 2‐years randomized controlled trial. European Journal of Clinical Nutrition, 65(9), 1048–1052. 10.1038/ejcn.2011.98 21629270

[fsn32420-bib-0006] Agrawal, R. P., Saran, S., Sharma, P., Gupta, R. P., Kochar, D. K., & Sahani, M. S. (2007). Effect of camel milk on residual beta‐cell function in recent onset type 1 diabetes. Diabetes Research and Clinical Practice, 77(3), 494–495. 10.1016/j.diabres.2007.01.012 17320238

[fsn32420-bib-0007] Agrawal, R. P., Swami, S. C., Beniwal, R., Kochar, D. K., Sahani, M. S., Tuteja, F. C., & Ghorui, S. K. (2003a). Effect of camel milk on glycemic control, lipid profile and diabetes quality of life in type 1 diabetes: A randomised prospective controlled cross over study. Indian Journal of Animal Sciences, 73(10), 1105–1110.

[fsn32420-bib-0008] Agrawal, R. P., Swami, S. C., Beniwal, R., Kochar, D. K., Sahani, M. S., Tuteja, F. C., & Ghouri, S. K. (2003b). Effect of camel milk on glycemic control, risk factors and diabetes quality of life in type‐1 diabetes: A randomised prospective controlled study. Journal of Camel Practice and Research, 10(1), 45–50. 10.1016/S0739-7240(03)00057-2

[fsn32420-bib-0009] Al‐Numair, K. S., Chandramohan, G., & Alsaif, M. A. (2011a). Effect of camel milk on collagen abnormalities in streptozotocin‐diabetic rats. African Journal of Pharmacy and Pharmacology, 5(2), 238–243. 10.1007/s12325-010-0098-2

[fsn32420-bib-0010] Al‐Numair, K. S., Chandramohan, G., & Alsaif, M. A. (2011b). Influence of camel milk on glycoprotein components in streptozotocin‐diabetic rats. Journal of Camel Practice and Research, 18(1), 15–20. 10.1186/1297-9716-42-72

[fsn32420-bib-0011] Andersen, E. S., Deacon, C. F., & Holst, J. J. (2018). Do we know the true mechanism of action of the DPP‐4 inhibitors? Diabetes, Obesity and Metabolism, 20(1), 34–41. 10.1111/dom.13018 28544214

[fsn32420-bib-0012] Ayoub, M. A., Palakkott, A. R., Ashraf, A., & Iratni, R. (2018). The molecular basis of the anti‐diabetic properties of camel milk. Diabetes Research and Clinical Practice, 146, 305–312. 10.1016/j.diabres.2018.11.006 30452940

[fsn32420-bib-0013] Badr, G., Ramadan, N. K., Sayed, L. H., Badr, B. M., Omar, H. M., & Selamoglu, Z. (2017). Why whey? Camel whey protein as a new dietary approach to the management of free radicals and for the treatment of different health disorders. Iranian Journal of Basic Medical Sciences, 20(4), 338–349. 10.22038/ijbms.2017.8573 28804604 PMC5425915

[fsn32420-bib-0014] Badr, G., Sayed, L. H., Omar, H.‐E.‐D.‐M., Abd El‐Rahim, A. M., Ahmed, E. A., & Mahmoud, M. H. (2017). Camel whey protein protects b and t cells from apoptosis by suppressing activating transcription factor‐3 (ATF‐3)‐mediated oxidative stress and enhancing phosphorylation of AKT and I kappa B‐alpha in type I diabetic mice. Cellular Physiology and Biochemistry, 41(1), 41–54. 10.1159/000455935 28142150

[fsn32420-bib-0015] Bailey, C. J., Tahrani, A. A., & Barnett, A. H. (2016). Future glucose‐lowering drugs for type 2 diabetes. The Lancet Diabetes and Endocrinology, 4(4), 350–359. 10.1016/s2213-8587(15)00462-3 26809680

[fsn32420-bib-0016] Beg, O. U., von Bahr‐Lindstrom, H. , Zaidi, Z. H., & Jornvall, H. (1986). A camel milk whey protein rich in half‐cystine. Primary structure, assessment of variations, internal repeat patterns, and relationships with neurophysin and other active polypeptides. European Journal of Biochemistry, 159(1), 195–201. 10.1111/j.1432-1033.1986.tb09852.x 3743571

[fsn32420-bib-0017] Beidokhti, M. N., & Jager, A. K. (2017). Review of antidiabetic fruits, vegetables, beverages, oils and spices commonly consumed in the diet. Journal of Ethnopharmacology, 201, 26–41. 10.1016/j.jep.2017.02.031 28257977

[fsn32420-bib-0018] Brezovecki, A., Cagalj, M., Dermit, Z. F., Mikulec, N., Ljoljic, D. B., & Antunac, N. (2015). Camel milk and milk products. Mljekarstvo, 65(2), 81–90. 10.15567/mljekarstvo.2015.0202

[fsn32420-bib-0019] Delzenne, N. M., Cani, P. D., Everard, A., Neyrinck, A. M., & Bindels, L. B. (2015). Gut microorganisms as promising targets for the management of type 2 diabetes. Diabetologia, 58(10), 2206–2217. 10.1007/s00125-015-3712-7 26224102

[fsn32420-bib-0020] Ejtahed, H. S., Naslaji, A. N., Mirmiran, P., Yeganeh, M. Z., Hedayati, M., Azizi, F., & Movahedi, A. M. (2015). Effect of camel milk on blood sugar and lipid profile of patients with type 2 diabetes: A pilot clinical trial. International Journal of Endocrinology and Metabolism, 13(1), e21160. 10.5812/ijem.21160 25745496 PMC4338669

[fsn32420-bib-0021] Galic, S., Oakhill, J. S., & Steinberg, G. R. (2010). Adipose tissue as an endocrine organ. Molecular and Cellular Endocrinology, 316(2), 129–139. 10.1016/j.mce.2009.08.018 19723556

[fsn32420-bib-0022] Heilbronn, L. K., Rood, J., Janderova, L., Albu, J. B., Kelley, D. E., Ravussin, E., & Smith, S. R. (2004). Relationship between serum resistin concentrations and insulin resistance in nonobese, obese, and obese diabetic subjects. The Journal of Clinical Endocrinology and Metabolism, 89(4), 1844–1848. 10.1210/jc.2003-031410 15070954

[fsn32420-bib-0023] Hu, C., & Jia, W. (2018). Diabetes in China: Epidemiology and genetic risk factors and their clinical utility in personalized medication. Diabetes, 67(1), 3–11. 10.2337/dbi17-0013 29263166

[fsn32420-bib-0024] Huang, Y., Yang, Z., Ye, Z., Li, Q., Wen, J., Tao, X., Chen, L., He, M., Wang, X., Lu, B., Zhang, Z., Zhang, W., Qu, S., & Hu, R. (2012). Lipocalin‐2, glucose metabolism and chronic low‐grade systemic inflammation in Chinese people. Cardiovascular Diabetology, 11, 11. 10.1186/1475-2840-11-11 22292925 PMC3295671

[fsn32420-bib-0025] Karlsson, F. H., Tremaroli, V., Nookaew, I., Bergstrom, G., Behre, C. J., Fagerberg, B., Nielsen, J., & Backhed, F. (2013). Gut metagenome in European women with normal, impaired and diabetic glucose control. Nature, 498(7452), 99–103. 10.1038/nature12198 23719380

[fsn32420-bib-0026] Khan, A. A., Alzohairy, M. A., & Mohieldein, A. H. (2013). Antidiabetic effects of camel milk in streptozotocin‐induced diabetic rats. American Journal of Biochemistry and Molecular Biology, 3(1), 151–158. 10.3923/ajbmb.2013.151.158

[fsn32420-bib-0027] Köchl, S., Niederstätter, H., & Parson, W. (2005). DNA extraction and quantitation of forensic samples using the phenol‐chloroform method and real‐time PCR. Methods in Molecular Biology, 297, 13–30. 10.1385/1-59259-867-6:013 15570097

[fsn32420-bib-0028] Korish, A. A. (2014). The antidiabetic action of camel milk in experimental type 2 diabetes mellitus: An overview on the changes in incretin hormones, insulin resistance, and inflammatory cytokines. Hormone and Metabolic Research, 46(6), 404–411. 10.1055/s-0034-1368711 24627103

[fsn32420-bib-0029] Law, I. K. M., Xu, A., Lam, K. S. L., Berger, T., Mak, T. W., Vanhoutte, P. M., Liu, J. T. C., Sweeney, G., Zhou, M., Yang, B., & Wang, Y. (2010). Lipocalin‐2 deficiency attenuates insulin resistance associated with aging and obesity. Diabetes, 59(4), 872–882. 10.2337/db09-1541 20068130 PMC2844835

[fsn32420-bib-0030] Leiherer, A., Muendlein, A., & Drexel, H. (2013). Phytochemicals and their impact on adipose tissue inflammation and diabetes. Vascular Pharmacology, 58(1–2), 3–20. 10.1016/j.vph.2012.09.002 22982056

[fsn32420-bib-0031] Mahmoud, M. H., Badr, G., & El Shinnawy, N. A. (2016). Camel whey protein improves lymphocyte function and protects against diabetes in the offspring of diabetic mouse dams. International Journal of Immunopathology and Pharmacology, 29(4), 632–646. 10.1177/0394632016671729 27694615 PMC5806827

[fsn32420-bib-0032] Martel, J., Ojcius, D. M., Chang, C.‐J., Lin, C.‐S., Lu, C.‐C., Ko, Y.‐F., Tseng, S.‐F., Lai, H.‐C., & Young, J. D. (2017). Anti‐obesogenic and antidiabetic effects of plants and mushrooms. Nature Reviews Endocrinology, 13(3), 149–160. 10.1038/nrendo.2016.142 27636731

[fsn32420-bib-0033] Mohamad, R. H., Zekry, Z. K., Al‐Mehdar, H. A., Salama, O., El‐Shaieb, S. E., El‐Basmy, A. A., Al‐said, M. G. A. M., & Sharawy, S. M. (2009). Camel milk as an adjuvant therapy for the treatment of type 1 diabetes: Verification of a traditional ethnomedical practice. Journal of Medicinal Food, 12(2), 461–465. 10.1089/jmf.2008.0009 19459752

[fsn32420-bib-0034] Mudgil, P., Kamal, H., Yuen, G. C., & Maqsood, S. (2018). Characterization and identification of novel antidiabetic and anti‐obesity peptides from camel milk protein hydrolysates. Food Chemistry, 259, 46–54. 10.1016/j.foodchem.2018.03.082 29680061

[fsn32420-bib-0035] Naveen, J., & Baskaran, V. (2018). Antidiabetic plant‐derived nutraceuticals: A critical review. European Journal of Nutrition, 57(4), 1275–1299. 10.1007/s00394-017-1552-6 29022103

[fsn32420-bib-0036] Newsholme, P., Keane, K. N., Carlessi, R., & Cruzat, V. (2019). Oxidative stress pathways in pancreatic beta‐cells and insulin‐sensitive cells and tissues: Importance to cell metabolism, function, and dysfunction. American Journal of Physiology‐Cell Physiology, 317(3), C420–C433. 10.1152/ajpcell.00141.2019 31216193

[fsn32420-bib-0037] Oh, K.‐J., Lee, D. S., Kim, W. K., Han, B. S., Lee, S. C., & Bae, K.‐H. (2017). Metabolic adaptation in obesity and type II diabetes: Myokines, adipokines and hepatokines. International Journal of Molecular Sciences, 18(1), 8. 10.3390/ijms18010008 PMC5297643 28025491

[fsn32420-bib-0038] Pedersen, B. K., & Febbraio, M. A. (2012). Muscles, exercise and obesity: Skeletal muscle as a secretory organ. Nature Reviews Endocrinology, 8(8), 457–465. 10.1038/nrendo.2012.49 22473333

[fsn32420-bib-0039] Rettedal, E. A., Altermann, E., Roy, N. C., & Dalziel, J. E. (2019). The effects of unfermented and fermented cow and sheep milk on the gut microbiota. Frontiers in Microbiology, 10, 458. 10.3389/fmicb.2019.00458 30930871 PMC6423907

[fsn32420-bib-0040] Rudkowska, I. (2009). Functional foods for health: Focus on diabetes. Maturitas, 62(3), 263–269. 10.1016/j.maturitas.2009.01.011 19233576

[fsn32420-bib-0041] Sboui, A., Djegham, M., Khorchani, T., Hammadi, M., Barhoumi, K., & Belhadj, O. (2010). Effect of camel milk on blood glucose, cholesterol and total proteins variations in alloxan‐induced diabetic dogs. International Journal of Diabetes and Metabolism, 18, 5–11. 10.1159/000497686

[fsn32420-bib-0042] Sboui, A., Khorchani, T., Djegham, M., Agrebi, A., Elhatmi, H., & Belhadj, O. (2010). Anti‐diabetic effect of camel milk in alloxan‐induced diabetic dogs: A dose‐response experiment. Journal of Animal Physiology and Animal Nutrition, 94(4), 540–546. 10.1111/j.1439-0396.2009.00941.x 19906135

[fsn32420-bib-0043] Schmitz, O., Brock, B., & Rungby, J. (2004). Amylin agonists: A novel approach in the treatment of diabetes. Diabetes, 53, S233–S238. 10.2337/diabetes.53.suppl_3.S233 15561917

[fsn32420-bib-0044] Steppan, C. M., Bailey, S. T., Bhat, S., Brown, E. J., Banerjee, R. R., Wright, C. M., Patel, H. R., Ahima, R. S., & Lazar, M. A. (2001). The hormone resistin links obesity to diabetes. Nature, 409(6818), 307–312. 10.1038/35053000 11201732

[fsn32420-bib-0045] Subbotina, E., Sierra, A., Zhu, Z. Y., Gao, Z., Koganti, S. R. K., Reyes, S., Stepniak, E., Walsh, S. A., Acevedo, M. R., Perez‐Terzic, C. M., Hodgson‐Zingman, D. M., & Zingman, L. V. (2015). Musclin is an activity‐stimulated myokine that enhances physical endurance. Proceedings of the National Academy of Sciences of the United States of America, 112(52), 16042–16047. 10.1073/pnas.1514250112 26668395 PMC4702977

[fsn32420-bib-0046] Thomas, R. L., Halim, S., Gurudas, S., Sivaprasad, S., & Owens, D. R. (2019). IDF diabetes atlas: A review of studies utilising retinal photography on the global prevalence of diabetes related retinopathy between 2015 and 2018. Diabetes Research and Clinical Practice, 157, 107840. 10.1016/j.diabres.2019.107840 31733978

[fsn32420-bib-0047] Tilg, H., & Moschen, A. R. (2014). Microbiota and diabetes: An evolving relationship. Gut, 63(9), 1513–1521. 10.1136/gutjnl-2014-306928 24833634

[fsn32420-bib-0048] Viciani, E. (2017). Genome watch carpe diet. Nature Reviews Microbiology, 15(11), 644. 10.1038/nrmicro.2017.123 29021595

[fsn32420-bib-0049] Wallace, T. M., Levy, J. C., & Matthews, D. R. (2004). Use and abuse of HOMA modeling. Diabetes Care, 27(6), 1487–1495. 10.2337/diacare.27.6.1487 15161807

[fsn32420-bib-0050] Wang, S. Y., Liang, J. P., Song, N. N., Shao, W. J., & Heng, H. (2009). Effect of raw camel milk in type 2 diapetes animal models and patients: Ten months randomised study. Journal of Camel Practice and Research, 16(1), 107–113. 10.1647/2008-037.1

[fsn32420-bib-0051] Wernery, U., Johnson, B., & Ishmail, W. T. (2006). Insulin content in raw dromedary milk and serum measured over one lactation period. Journal of Camel Practice and Research, 13(2), 89–90. 10.1016/j.jtcvs.2004.01.015

[fsn32420-bib-0052] Wiegel, J., Tanner, R., & Rainey, F. A. (2006). An introduction to the family *Clostridiaceae* . The prokaryotes (3rd edn., vol. 4, pp.654–678). New York, NY: Springer. 10.1007/0-387-30744-3_20

[fsn32420-bib-0053] Zhang, X., Zhao, Y., Xu, J., Xue, Z., Zhang, M., Pang, X., Zhang, X., & Zhao, L. (2015). Modulation of gut microbiota by berberine and metformin during the treatment of high‐fat diet‐induced obesity in rats. Scientific Reports, 5, 14405. 10.1038/srep14405 26396057 PMC4585776

[fsn32420-bib-0054] Zhu, Y.‐S., Gu, Y., Jiang, C., & Chen, L. (2019). Osteonectin regulates the extracellular matrix mineralization of osteoblasts through P38 signaling pathway. Journal of Cellular Physiology, 235(3), 2220–2231. 10.1002/jcp.29131 31489629

