# Supplementary figures and images for: Hypoglycemic effect of camel milk powder in type 2 diabetic patients: A randomized, double‐blind, placebo‐controlled trial
Source: Food Sci Nutr. 2021 Jun 29;9(8):4461–72. doi: 10.1002/fsn3.2420 (PMC8358379; doi:10.1002/fsn3.2420)

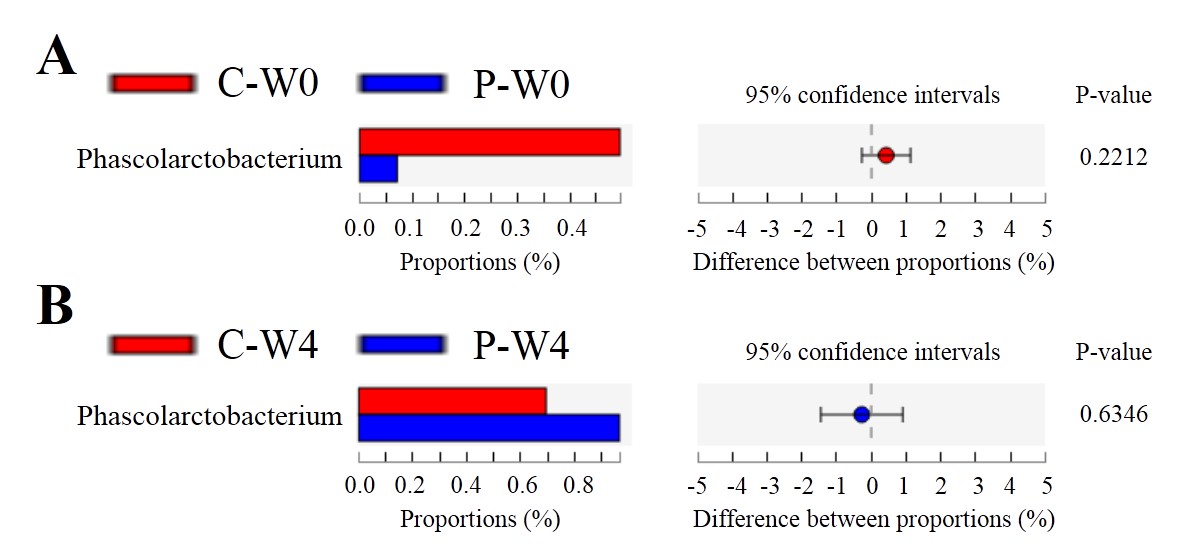

Supplement: Supplementary file 1 — Figure S1 [file FSN3-9-4461-s001.jpg]
